# Circumventricular Organs and Parasite Neurotropism: Neglected Gates to the Brain?

**DOI:** 10.3389/fimmu.2018.02877

**Published:** 2018-12-11

**Authors:** Marina Bentivoglio, Krister Kristensson, Martin E. Rottenberg

**Affiliations:** ^1^Department of Neuroscience Biomedicine and Movement Sciences, University of Verona, Verona, Italy; ^2^Department of Neurosciences, Karolinska Institutet, Stockholm, Sweden; ^3^Department Microbiology, Tumor and Cell Biology, Karolinska Institutet, Stockholm, Sweden

**Keywords:** choroid plexus, *Trypanosoma brucei*, immune responses, brain infections, sleep disorders, lymphocytic choriomeningitis virus, circadian rhythms, blood-brain barrier

## Abstract

Circumventricular organs (CVOs), neural structures located around the third and fourth ventricles, harbor, similarly to the choroid plexus, vessels devoid of a blood-brain barrier (BBB). This enables them to sense immune-stimulatory molecules in the blood circulation, but may also increase chances of exposure to microbes. In spite of this, attacks to CVOs by microbes are rarely described. It is here highlighted that CVOs and choroid plexus can be infected by pathogens circulating in the bloodstream, providing a route for brain penetration, as shown by infections with the parasites *Trypanosoma brucei*. Immune responses elicited by pathogens or systemic infections in the choroid plexus and CVOs are briefly outlined. From the choroid plexus trypanosomes can seed into the ventricles and initiate accelerated infiltration of T cells and parasites in periventricular areas. The highly motile trypanosomes may also enter the brain parenchyma from the median eminence, a CVO located at the base of the third ventricle, by crossing the border into the BBB-protected hypothalamic arcuate nuclei. A gate may, thus, be provided for trypanosomes to move into brain areas connected to networks of regulation of circadian rhythms and sleep-wakefulness, to which other CVOs are also connected. Functional imbalances in these networks characterize human African trypanosomiasis, also called sleeping sickness. They are distinct from the sickness response to bacterial infections, but can occur in common neuropsychiatric diseases. Altogether the findings lead to the question: does the neglect in reporting microbe attacks to CVOs reflect lack of awareness in investigations or of gate-opening capability by microbes?

## Introduction

Parasites can attack the central nervous system (CNS) through the blood, with the olfactory route used by certain amoebas such as *Naegleria fowleri* as remarkable exceptions (1). Invasion from the bloodstream through CNS vessels is hampered by the blood-brain barrier (BBB) that restricts non-selective transcytosis of molecules across the endothelial cells, which are linked by tight junctions. Certain parasites, such as *Toxoplasma*, have developed mechanisms to cross the BBB (2, 3), while others (*Taenia, Schistosoma, Plasmodium*) do not traverse the BBB but induce inflammation in the surrounding parenchyma if trapped in vessels (4–7).

Another hematogenous route for parasite entry into the brain is via the choroid plexus (CP) and circumventricular organs (CVOs) though this route is largely neglected. These structures are devoid of a BBB, but a blood- cerebrospinal fluid (CSF) barrier is formed by tight junctions between the CP epithelial cells and between specialized tanycytes which outline the CVOs (8).

The present overview highlights that parasites can target the CP and CVOs. The role of CP as initiator of brain infections and inflammation is briefly outlined. Focus is then on the “minute” CVOs as sites of direct parasite attacks. This is exemplified by the extracellular parasites *Trypanosoma brucei* (*T. b*.), the etiological agents of African trypanosomiasis, that could cause distinct brain homeostatic imbalances by their localization in the CVOs.

## Which are the CVOs?

**Figure 1:**
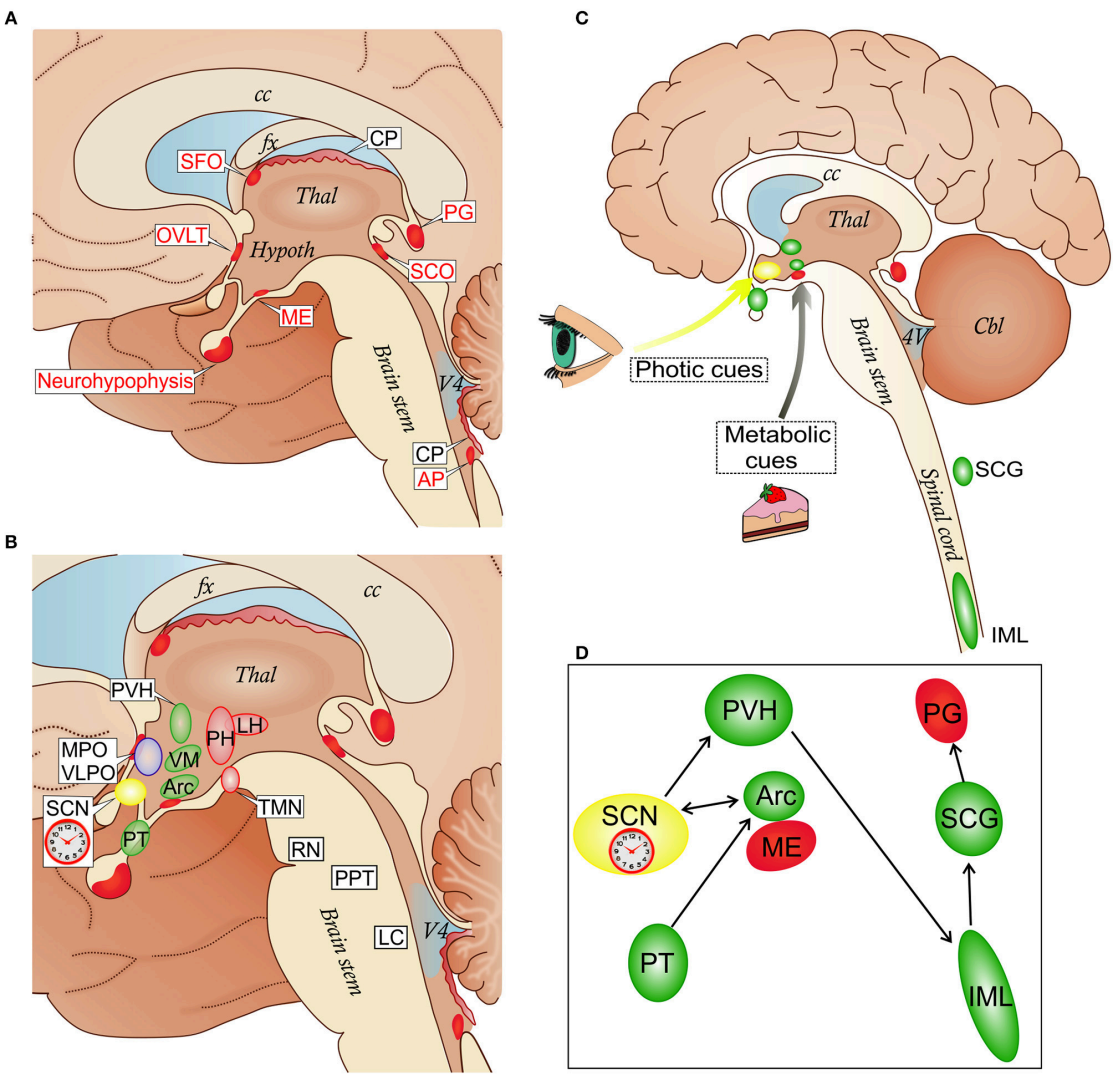
**(A)** Localization of circumventricular organs (CVOs) and choroid plexus (CP) in a schematic view of the medial brain surface. The depicted CVOs play homeostatic roles by their secretory and sensory functions. The neurohypophysis, the *distal part* (the neural lobe), receives the hypothalamic neurohormones vasopressin and oxcytocin secreted into the superficial vascular plexus in the *proximal part*. The deep vascular plexus of the median eminence (ME) serves a sensory function mediating metabolic signals to the arcuate nucleus (Arc) of the hypothalamus (see also **C**). The organum vasculosum of the lamina terminalis (OVLT) participates in osmoregulation by controlling vasopressin release and in the control of the cyclic production of the hypophysial gonadotropic hormones. The subfornical organ (SFO) participates in the regulation of vasopressin release and induction of the sensation of thirst caused by dehydration. The subcommissural organ (SCO) may facilitate the flow of the cerebrospinal fluid in the cerebral aqueduct between the third and fourth ventricles. The pineal gland (PG) secretes melatonin and stabilizes the photoperiod timekeeping. The area postrema (AP) is involved in cardiovascular and respiratory regulation, as well as in the control of the vomiting center in the medulla (8). **(B)** CVOs and key nodes in the regulation of sleep, wakefulness and their circadian alternation: the biological clock which resides in the suprachiasmatic nucleus (SCN); wake-promoting cell groups in the posterior hypothalamus (PH) which include the histamine-containing tuberomammillary nucleus (TMN), and the lateral hypothalamus (LH) where orexin neurons are located; sleep-promoting cell groups in the anterior hypothalamus (MPO, medial preoptic area; VPLO, ventrolateral preoptic nucleus). Other sleep-wake-regulatory cell groups are depicted in the brain stem: serotonergic raphe nuclei (RN), cholinergic cells of the pedunculopontine tegmentum (PPT) and noradrenergic neurons of the locus coeruleus (LC). In the hypothalamus, the CVOs network involves the paraventricular nucleus (PVH), ventromedial nucleus (VM), and pars tuberalis (PT); the Arc forms an anatomical and functional complex with the ME. **(C,D)** Schematic representation of the integration of the ME/Arc complex in the circadian rhythm-generating system. ME and Arc neurons are exposed to metabolic cues. This influences the semi-autonomous clock in the Arc, which has reciprocal connections with the master clock in the SCN entrained mainly by photic cues (9, 10). The SCN is connected via the PVH, spinal cord (sympathetic intermediolateral column, IML) and superior cervical ganglia (SCG) to the PG, in a circuit which regulates melatonin secretion. The SCG is also a site for early trypanosome invasion (11). Melatonin secreted from the PG exerts a feedback regulation through melatonin receptor binding sites in the SCN and PT in the mediobasal hypothalamus which is connected to the Arc (12). cc, corpus callosum; fx, fornix; Thal, thalamus; Hypoth, hypothalamus; V4, fourth ventricle.

The term CVO was coined to designate structures located around the third and fourth brain ventricles including, in mammals, the pineal gland, subcommissural organ, median eminence (ME), neurohypophysis, and organum vasculosum of the lamina terminalis (OVLT), as well as area postrema (AP) and subfornical organ (SFO) (Figure 1A) (13). The phylogenetic origins of CVOs do not suggest any unifying ancestral program, and cross-species comparisons suggest a high degree of evolutionary flexibility (14). In spite of this, the CVOs, visualized by molecular tracers excluded by the BBB, have a number of common features (8): they are located at the midline in the ventricular walls and are sealed off from the CSF by elongated tanycytes (15). With the exception of the rodent subcommissural organ, in which vessels are sparse, they are highly vascularized and capillaries are endowed with small fenestrations (about 60 nm), which are bridged by a thin diaphragm formed by radial fibrils surrounding a slit-shaped pore (about 5 nm) (16–18). In OVLT, AP and SFO the capillaries are surrounded by large perivascular spaces delineated by an outer laminin-containing basement membrane (BM), which impede diffusion of dextrans larger than about 10 kD into the parenchyma (19, 20).

The location of CVOs at the interface between brain tissues, bloodstream and CSF indicates a role in interactions between these compartments and in homeostatic regulations. The OVLT, AP, and SFO have sensory functions, responding to blood-borne signals, while the neurohypophysis/ME and pineal gland are secretory, releasing neurohormones into blood vessels (8, 21). Of note, the ME also serves a sensory function, playing a major role in the regulation of metabolic signals that control energy balance. For instance, blood levels of the adipose tissue-derived hormone leptin, which inhibits feeding, are sensed by leptin receptors at either dendritic-like neuronal processes (22) or tanycytes (23). The former are derived from the hypothalamic arcuate nucleus (Arc), which is sealed by the BBB, and pass the border between Arc and ME (21).

## Immune Responses in the CP and CVOs

The CP, where the CSF is formed, does not contain neurons and, although originally considered as a CVO (13), is often not included in this group of structures (8). The CP also has fenestrated capillaries and is lined by specialized epithelial cells that isolate it from the ventricles. It can be target of microbial attacks, and may contribute to spread of infections to the CNS.

A role for the CP in initiating T cell invasion of the brain parenchyma was suggested by experimental autoimmune encephalomyelitis (EAE), a most studied paradigm of brain inflammation. In EAE, Th1 and Th17 cells can enter the brain parenchyma crossing the BBB at postcapillary venules by first traversing endothelial cells and BM to enter perivascular spaces, where antigen-presenting cells may be located (24, 25). T cells activated by antigen recognition may then enter the parenchyma after passing the next hindrance, the astrocytic BM (26). This two-step passage may be triggered by CP-derived Th17 cells, which could reach perivascular spaces via the CSF flow from the ventricles and subarachnoid space (27).

Lymphocytic choriomeningitis virus (LCMV) is the prototype of a non-cytolytic virus that causes immune-mediated brain diseases (28). In adult mice, LCMV infects meningeal cells, CP epithelial cells and ependymal cells, and causes T cell infiltration in the meninges and CP (29). This is followed by infection of resident cells and T cell invasion in periventricular regions and white matter (30). The brain invasion of T cells is triggered by activation of viral pattern-recognition receptors on resident cells [probably microglia (28)] that secrete interferon (IFN)-α/β, which initiates production of the chemokine CXCL10 by astrocytes (31). This production is markedly increased by IFN-γ once virus-sensitized T cell are recruited, accelerating the inflammatory response (31). A similar two-phase brain invasion of T cells is seen during infection with the parasite *T. b. brucei* (32), as described below.

Knowledge on the activation of CVOs during systemic infections, which is raising increasing interest, has been based on the effects of the bacterial cell wall product lipopolysaccharide (LPS) (33). This molecule binds Toll-like receptor (TLR)4, which is expressed in the CVOs (34). LPS activation of non-hematopoietic, resident CVO cells can elicit sustained CNS-specific inflammation independent of cytokine effects (35). LPS can also cause robust proliferation of resident microglia in the CVOs and neighboring hypothalamus (36).

Signs of microglia activation in OVLT, SFO, ME, and AP are observed even in normal, unchallenged, adult mice, and this may reflect a continuous exposure to molecules from the bloodstream (37). Interestingly, microglial signaling in the CVOs of the mediobasal hypothalamus can regulate the control of energy homeostasis (38).

## CP and Parasite Infections

Information on the CP as target of parasite infections is sparse. *Toxoplasma gondii* tachyzoites and pseudocysts were seen in the CP epithelium and stroma in the majority of AIDS patients deceased during acute necrotizing stages of cerebral toxoplasmosis, and in 20% of the patients with healed lesions (39). A blood-borne spread from reactivated systemic infections with a potential for further CSF dissemination was suggested (39). In *Toxoplasma*-infected mice, however, no significant inflammation was seen in the CP, arguing for a preferential recruitment of leukocytes across the cerebral blood vessels to control the spread of infection (40).

Sporadic reports indicating that parasites, in addition to some bacteria, can infect the CP include *Schistosoma mansoni*, which may move to the CP and shed eggs for further spread into the CNS, and *Toxocara canis*, which may reach the CP following systemic infections (41).

The involvement of the CP in parasitic infections has been most studied in *T. b*. infections. Neuropathological investigations of victims of human African trypanosomiasis (HAT) have shown infiltration of white blood cells in the brain parenchyma, preferentially around the third ventricle and in the white matter, i.e., “leukoencephalitis” (42), indicating that this event follows an attack of trypanosomes to the CP.

Early experimental studies employing *T. b*. sub-species have revealed indeed trypanosomes in the CP of infected monkeys (43, 44) and dogs (45, 46). The localization of trypanosomes to the CP was verified using immunohistochemistry in rodents (11) and by electron microscopy, which showed parasites penetrating the CP vessels and epithelial cell layer to reach the CSF in the ventricles (47).

From the ventricular CSF a few trypanosomes may invade the ependymal cell layer (48) and spread into the surrounding brain parenchyma (hypothalamus, white matter). An immune response is later initiated at pericapillary spaces that trigger passage of T cells across the BBB by mechanisms similar to those occurring in EAE and in LCMV infections described above. The T cells open the way for a new and stronger wave of trypanosome invasion into the brain, which may occur weeks following the CP infection (49–51). As in LCMV infections, the process is amplified by T cell-derived release of IFN-γ and by astrocytic expression of CXCL10 (49, 51, 52).

Infections with *T. b. brucei*, thus, show striking similarities with LCMV infections, not only in spatial localization to periventricular and deeper brain structures, but also in immune responses, which during infections with the extracellular trypanosomes play a paradoxical role of enhancing pathogen neuroinvasion. Trypanosome infections do not spread into superficial neocortical layers from the meninges (47, 49) and, by affecting the CP, pathogen and immune cell attacks to the brain parenchyma can instead be launched from within the brain.

## Trypanosome Attack to the CVOs

Knowledge on the attack of neurotropic parasites to the CVOs rely on data obtained with experimental *T. b. brucei* rodent infections. Of note, trypanosomes target the CVOs already early during the infection when parasites have not yet crossed the vessels endowed with tight junctions located in the brain and retina (11), and a local immune response is elicited (53, 54). This response includes expression of TNF-α, interleukin (IL)-1β, IL-1β converting enzyme and inhibitory factor I_κ_Bα mRNAs in the ME/Arc complex, OVLT and AP (53), as well as T cell invasion (54).

The early infection of CVOs by trypanosomes is of interest in view of pathogenetic mechanisms of neural dysfunction. In HAT, which is also called sleeping sickness, functional disturbances include a fragmented sleep pattern and alterations of sleep architecture, documented also in experimental infections in rats (55), and a disruption of the sleep-wake cycle, which is a major circadian rhythm, leading to diurnal somnolence and nocturnal insomnia (56). Other circadian rhythms are also disrupted in HAT patients (56). Functional disturbances worsen progressively during the disease.

Experimental findings have indicated that typical alterations of sleep architecture due to dysfunction of hypothalamic neurons can initiate when parasites have not yet invaded the brain parenchyma, as discussed below. The early trypanosome infection of the CVOs could therefore contribute to triggering initial and distinct CNS imbalances.

### CVOs and the Networks of Sleep-Wake and Circadian Regulation

Major alterations of sleep architecture (the so-called “sleep-onset rapid eye movement episodes”) can represent early signs of HAT (56) and they can precede parasite traversal of the BBB as seen in experimental rat infections (57).

These distinct sleep changes are due to altered function of the population of neurons which release the orexin/hypocretin peptide pair and reside in the lateral hypothalamus (LH) [review, see (58)]. Orexin neurons, which are important for energy homeostasis, wakefulness stability and transitions between vigilance states, are especially sensitive to inflammatory signaling due to their molecular structure (58). Damage and dysfunction of orexin neurons during the progression of *T. b*. infection have been documented in experimental models (57, 59).

However, in the early stage of trypanosome infection, proinflammatory molecules from the CVOs could potentially affect the function of orexin neurons through both humoral and neural pathways. The OVLT, AP, SFO are directly connected with the LH (60), and orexin axons densely innervate the Arc (61), receiving reciprocal connections. Several neural pathways connect the CVOs with other sleep-wake-regulatory hypothalamic structures, such as the preoptic area (60, 62) (Figure 1B).

It is also important to note that the distributed system of regulation of sleep architecture and sleep-wake cycle has key centers in the hypothalamus and brainstem in proximity to the third and fourth ventricles [review, see (58)], and that the master circadian pacemaker, the suprachiasmatic nucleus (SCN) (63) resides in the anterior hypothalamus, ventral to the third ventricle (Figure 1B). Projections of the SFO to the SCN and polysynaptic pathways linking OVLT and AP with the SCN have been reported (64–66). Importantly, Arc neurons project to the SCN, transmitting feeding-related signals to the master biological clock (67) (Figures 1B,C).

### Trypanosomes and the ME-Arc Complex

**Figure 2:**
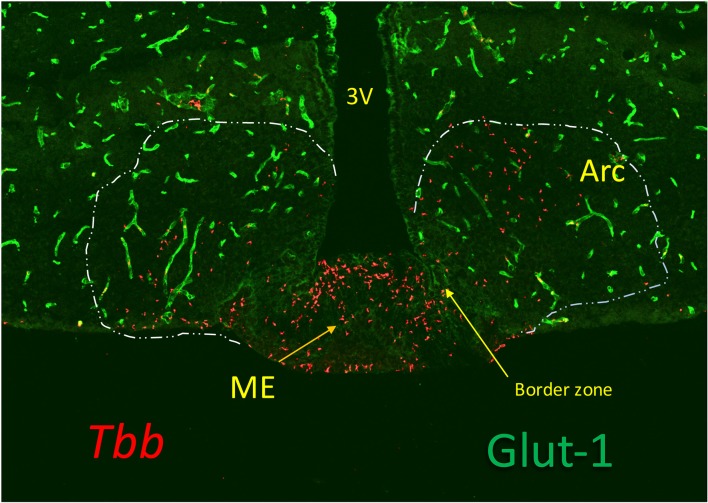
Trypanosomes (*T. b. brucei*; red) invasion of the median eminence (ME), which lacks Glut-1, a glucose transporter expressed in the cerebral vessels (green). The image shows that the parasites locate at the vascular layer at the bottom of the ME and at the floor of the third ventricle (3V) where the tanycytes are linked by tight junctions. They are also found in the arcuate nucleus (Arc), sealed by the blood-brain barrier (BBB) of the cerebral vessels (green), probably bypassing the border zone in which tanycytes are linked by adherens junctions. Although the vascular layer contains fenestrated capillaries, trypanosomes (and leukocytes) are much too large to pass through the fenestrae (60 nm) or their slit-sized pores (5 nm), and they probably, like in other tissues, pass across the postcapillary venules that in ME lack a BBB. Extension of the fenestrated capillaries in the ME is flexible, and can be stimulated by metabolic cues (73), but whether this is affected by inflammatory responses is not known.

Early trypanosome invasion of the Arc from the ME has been observed in infected rats (57). Dividing slender trypanosomes are highly motile (68) and the motility is driven by rotational flagellar movements, like drills, critical for virulence (69). Trypanosomes can also change directions not to get trapped, which favors movements in tissues (70). These parasites can therefore move in the ME from the vessels into the layer of tanycytes, and such progression may be facilitated by parasite-derived proteases (71, 72). A gate to pass toward Arc neurons may be provided to trypanosomes by the border between ME and Arc (21) (Figure 2).

By infiltrating the ME/Arc complex trypanosomes may affect not only neural mechanisms of sleep regulation and circadian rhythm generation, but also other physiological functions. For instance, HAT is associated with reproductive disorders (74). In *T. congolense*-infected goats a failure in luteinizing hormone release is due to a disruption in secretion of the gonadotropin-releasing hormone into ME vessels (75). Whether secretion of other hormones into ME vessels (76) or energy metabolism regulated by the ME are also disturbed in trypanosome infections remains to be studied.

### Trypanosomes and the Pineal Gland

Melatonin, the hormone produced by the pineal gland, is secreted with a circadian rhythm and can act as an endogenous circadian rhythm synchronizer to stabilize or reinforce circadian rhythms (77). Clinical studies on HAT patients have shown that the circadian rhythm of melatonin plasma level is maintained, but with a significant phase-advance in its peak, which is nocturnal (78). In *T. b. brucei*-infected rats the binding of melatonin to its receptor in the SCN, which entrains the circadian rhythm of melatonin secretion to photic stimuli (Figures 1C,D), is phase-advanced (79) and a melatonin agonist can restore synchronized sleep fragmentation during the infection (80).

Taken together, these observations indicate that disturbances in the melatonin-generating system play a pathogenetic role for sleep fragmentation in African trypanosomiasis.

### Which Mechanisms May Induce Functional Imbalances Upon Trypanosome Attack to the CVOs?

Stimulation with LPS and its effector molecules IL-1β and TNF is associated with “sickness behavior” dominated by hypersomnia and fever. From the functional point of view it is important to recall that somnolence during sickness behavior does not have any of the distinct changes of sleep architecture, sleep-wake cycle and circadian rhythms that characterize African trypanosomiasis [review, see (58)] and are also akin to those in certain neuropsychiatric illnesses (81). Mechanisms of functional imbalance caused by trypanosome attack to the CVOs should therefore be different from those elicited by bacterial LPS.

Trypanosomes release a number of molecules that can act as immune stimulants (82), most notably the variable surface glycoprotein (VSG) and CpG-DNA, which is released by dead trypanosomes and activates TLR9 signaling (83). No information is available on the effects of VSG on the brain. Systemic administration of bacterial CpG-oligonucleotides activates cells in CVOs as well as in the hypothalamic paraventricular nucleus (as revealed by the neuronal activity markers p-STAT3 and Fos). Activation of TLR4 and TLR9 converge on the MyD88 intracellular signaling pathways, but with some differences in their effects (84). Interestingly, TLR9 is expressed on neuronal subsets in the brain, but its role is still unclear (85).

CVOs harboring trypanosomes may also be exposed to prostaglandins (PG) synthesized by the parasites (86). Of these, PGE2 could be involved in dysfunction characteristic of African trypanosomiasis. PGE2 has limited effects on LPS-induced hypersomnia (87), but can cause phase shifts in peripheral circadian clocks and does not affect locomotor activity rhythm, which is SCN-dependent (88). This is similar to observations in *T. b. brucei*-infected rats in which locomotor rhythm is maintained, while the circadian rhythm in clock gene expression in CVOs is disturbed (89). Interestingly, it has been recently reported in mice that trypanosomes release also a soluble factor, still unidentified, that causes shortening of the circadian peak periods (90).

These data sets indicate that trypanosome attack to the CVOs elicits immune signaling mechanisms that in intensity, timing or quality differ from those of systemic bacterial infections.

## CVOs in Viral and Prion Infections

To our knowledge, infections of CVOs with microbes have not been reported in humans. Experimental observations on viruses are rare, but include LCMV (91), bat rabies strains (92), and Venezuelan Western equine virus (93) infections. Remarkable exceptions are, however, prion infections. This pathogen can spread to the brain via peripheral and autonomic nerve fibers innervating the oral-gastro-intestinal tract (60). At preclinical stages of the disease in sheep, prion immunolabeling is found in CVOs (AP, ME, SFO, OVLT, pineal gland and neurohypophysis) at the same time as in the dorsal motor nucleus of the vagus nerve, and before prion spread into the surrounding tissues (94). The functional significance of prion localization to CVOs is not clear, but prions can directly affect synaptic functions and elicit anti-inflammatory immune responses (95).

## Concluding Remarks

The ring formed by CVOs around the third and fourth ventricles can be seen as a hub where systemic immune responses and homeostatic brain networks interact (96). These networks regulate metabolism, energy balance, neurohormonal secretion, as well as sleep, wakefulness, their alternation and other endogenous circadian rhythms. The response of CVOs to systemic bacterial innate receptor agonists and their role in sickness behavior have received attention, but direct infections of the CVOs have been neglected. This raises key questions:

- Do trypanosomes have peculiar properties to promote their spread to the CVOs, or is the lack of information on direct infection of the CVOs by other microbes a consequence of lack of proper studies? Systematic neuropathological studies and application of new imaging techniques (97) are needed to answer this question.
- Does the targeting of trypanosomes to the CVOs elicit a spectrum of immune signals distinct in intensity or quality from those elicited by bacterial LPS in systemic infections?
- Could the CVOs, defined as “windows of the brain” (98) implying open windows of the BBB, actually represent “gates to the brain” that only motile microbes have learned how to open and other microbes may access by different mechanisms? This would require a targeted fight against microbes sitting in the CVOs, and solicits attention on the infection of the minute organs surrounding the ventricles.

## Author Contributions

All authors listed have made a substantial, direct and intellectual contribution to the work, and approved it for publication.

### Conflict of Interest Statement

The authors declare that the research was conducted in the absence of any commercial or financial relationships that could be construed as a potential conflict of interest.
